# A Novel Necroptosis-Associated IncRNAs Signature for Prognosis of Head and Neck Squamous Cell Carcinoma

**DOI:** 10.3389/fgene.2022.907392

**Published:** 2022-06-08

**Authors:** Jing Huang, Rong Lu, Dongta Zhong, Youliang Weng, Lianming Liao

**Affiliations:** ^1^ Department of Pharmacy, Fujian Medical University Cancer Hospital and Fujian Cancer Hospital, Fuzhou, China; ^2^ Department of Laboratory Medicine, The First Affiliated Hospital of Xiamen University, Xiamen Key Laboratory of Genetic Testing, School of Medicine, Xiamen University, Xiamen, China; ^3^ Department of Medical Oncology, Union Hospital of Fujian Medical University, Fuzhou, China; ^4^ Department of Radiation Oncology, Fujian Medical University Cancer Hospital and Fujian Cancer Hospital, Fuzhou 350014, China; ^5^ Center of Laboratory Medicine, Union Hospital of Fujian Medical University, Fuzhou, China

**Keywords:** risk score, prognosis, squamous cell carcinoma, necroptosis, immune, tumor

## Abstract

**Purpose:** The prognosis of head and neck squamous cell carcinoma (HNSCC) is poor. Necroptosis is a novel programmed form of necrotic cell death. The prognostic value of necroptosis-associated lncRNAs expression in HNSCC has not been explored.

**Methods:** We downloaded mRNA expression data of HNSCC patients from TCGA databases. Prognostic lncRNAs were identified by univariate Cox regression. LASSO was used to establish a model with necroptosis-related lncRNAs. Kaplan-Meier analysis and ROC were applied to verify the model. Finally, functional studies including gene set enrichment analyses, immune microenvironment analysis, and anti-tumor compound IC50 prediction were performed.

**Results:** We identified 1,117 necroptosis-related lncRNAs. The Cox regression showed 55 lncRNAs were associated with patient survival (*p* < 0.05). The risk model of 24- lncRNAs signature categorized patients into high and low risk groups. The patients in the low-risk group survived longer than the high-risk group (*p* < 0.001). Validation assays including ROC curve, nomogram and correction curves confirmed the prediction capability of the 24-lncRNA risk mode. Functional studies showed the two patient groups had distinct immunity conditions and IC50.

**Conclusion:** The 24-lncRNA model has potential to guide treatment of HNSCC. Future clinical studies are needed to verify the model.

## Introduction

Head and neck squamous cell carcinomas (HNSCCs) arise from squamous cells in the oral cavity, pharynx and larynx. The most common risk factors for HNSCC include alcohol drinking, smoking and HPV infection (Fakhry et al., 2008). Athough HNSCC can be treated with surgery, radiotherapy and chemotherapy, patients with HNSCCs still suffer from poor survival. To improve patient survival, novel therapeutic targets and effective prognostic tools are needed.

Necroptosis is another mode of regulated cell death mimicking apoptosis and necrosis. Necroptosis is associated with a range of pathological conditions and diseases, including cancer. It is mediated by Fas, TNF, LPS, and death receptors (Vanden Berghe et al., 2014). Binding of ligands and receptors activates RIP3, which phosphorylates MLKL (Sun et al., 1999). Phosphorylated MLKL then translocates to and ruptures cellular membranes, leading to cell swelling and release of intracellular components (Dondelinger et al., 2014; Hildebrand et al., 2014; Wang et al., 2014).

A plethora of evidence shows necroptosis of tumor cells is often associated with tumor aggressiveness and metastasis. RIP3, a molecular marker of necroptosis, is an independent factor associated with survival in breast cancer (Koo et al., 2015). RIP3 expression was also decreased in colorectal cancer and was an independent prognostic factor of survival (Feng et al., 2015). In acute myeloid leukemia, RIP3 expression was reduced in most samples and overexpression of RIP3 in DA1-3b leukemia cells induced necroptosis (Nugues et al., 2014). Li et al. reported that necroptosis was associated with survival of HNSCC patients (Li et al., 2020).

Long non-coding RNAs (lncRNAs) regulate gene expression and are involved in tumorogenesis (Kumar and Goyal, 2017; Peng et al., 2017). Specially, Jiang et al. reported dysregulation of lncRNAs was involved in HNSCC(Jiang et al., 2019). Although necroptosis plays an important role in patient survival of a variety of tumors, the role of necroptosis-related lncRNAs in HNSCC has not been reported.

We thus explored the potential roles of different necroptosis-related lncRNAs on the survival of HNSCC patients. We developed a novel risk-score model with necroptosis-related lncRNAs according to their expression levels. The results might further our understanding of necroptosis in HNSCC.

## Materials and Methods

### TCGA Data Acquisition

The Cancer Genome Atlas (TCGA) (https://portal.gdc.cancer.gov/repository) has transcriptomic data of more than 20,000 cancer and normal samples. In the present study RNA sequencing (RNA-seq) data of tumor tissues of 487 HNSCC patients and 42 matched normal tissues was downloaded from TCGA database. Our study was conducted by reviewing public database and ethical approval was not required.

### Identification of Necroptosis-Related lncRNAs

The expression data of 67 necroptosis-associated genes was used for analysis (Supplementary Table S1). Correlation analysis was performed among 67 necroptosis-related genes and differentially expressed lncRNAs in the combined matrices. 1,117 lncRNAs with Pearson correlation coeffi-cients >0.4 and *p* < 0.001 were identified to be necroptosis-related lncRNAs.

### Establishment and Validation of the Risk Signature

The clinical data of HNSCC patients was downloaded from TCGA data portal. The univariate Cox proportional hazard regression analysis was used to screen prognostic genes. Least absolute shrinkage and selection operator (Lasso) regression was conducted with 10-fold cross-validation and a *p* value of 0.05. After identification of the prognostic lncRNAs, the risk scores were determined as follow (X: coefficients, Y: expression level of lncRNAs):

$$\text{r}\text{i}\text{s}\text{k}\;\text{s}\text{c}\text{o}\text{r}\text{e}=\sum_{i}^{n}Xi*Yi$$

HNSCC patients were allocated into either low- or high-risk groups according to the median risk score (Meng et al., 2019; Hong et al., 2020). The Chi-square test was used to determine the prognostic significance value of the risk model, and overall survival (OS) time was compared between the two groups *via* Kaplan-Meier analysis. The “survival”, “survminer” and “timeROC” R packages were used to plot the 1-, 3-, and 5-years receiver operating characteristics (ROC) curves. The risk scores were also evaluated as an independent risk factor with other clinical parameters by Cox regression with rms R package. Then a nomogram for prediction of the 1-, 3-, and 5-years OS was set up using risk score and clinical parameters. The ROC, calibration curves and Hosmer-Lemeshow test of the nomogram were assessed in the validation set.

### GSEA

To explore the biological pathways that might be responsible for poor patient survival, we employed R (Bioconductor package gsea) to perform gene set enrichment analyses (GSEA). Potential biological mechanisms of the prognostic model were also explored. KEGG gene sets in the GSEA database were downloaded. We chose gene sets with a FDR value < 0.05 and a FDR <0.25.

### The Investigation of the TME and Immune Checkpoints

CIBERSORT, EPIC, MCPcounter, QUANTISEQ, TIMER, and XCELL were used to evaluate cells in the tumor microenvironment (TME) (http://timer.cistrome.org/). ggplot2, ggtext, limma, and scales R packages and Wilcoxon signed-rank test were performed to analyze cell types in TME (Hong et al., 2020). TME scores and immune checkpoint were compared between the two groups with ggpubr R package.

### Prediction of Clinical Treatment Response

To predict therapeutic response, the R package pRRophetic was utilized to measure the half-maximal inhibitory concentrations (IC50) of each HNSCC sample on Genomics of Drug Sensitivity in Cancer (GDSC) (https://www.cancerrxgene.org/) (Geeleher et al., 2014).

## Results

### Extraction of lncRNAs

**FIGURE 1:**
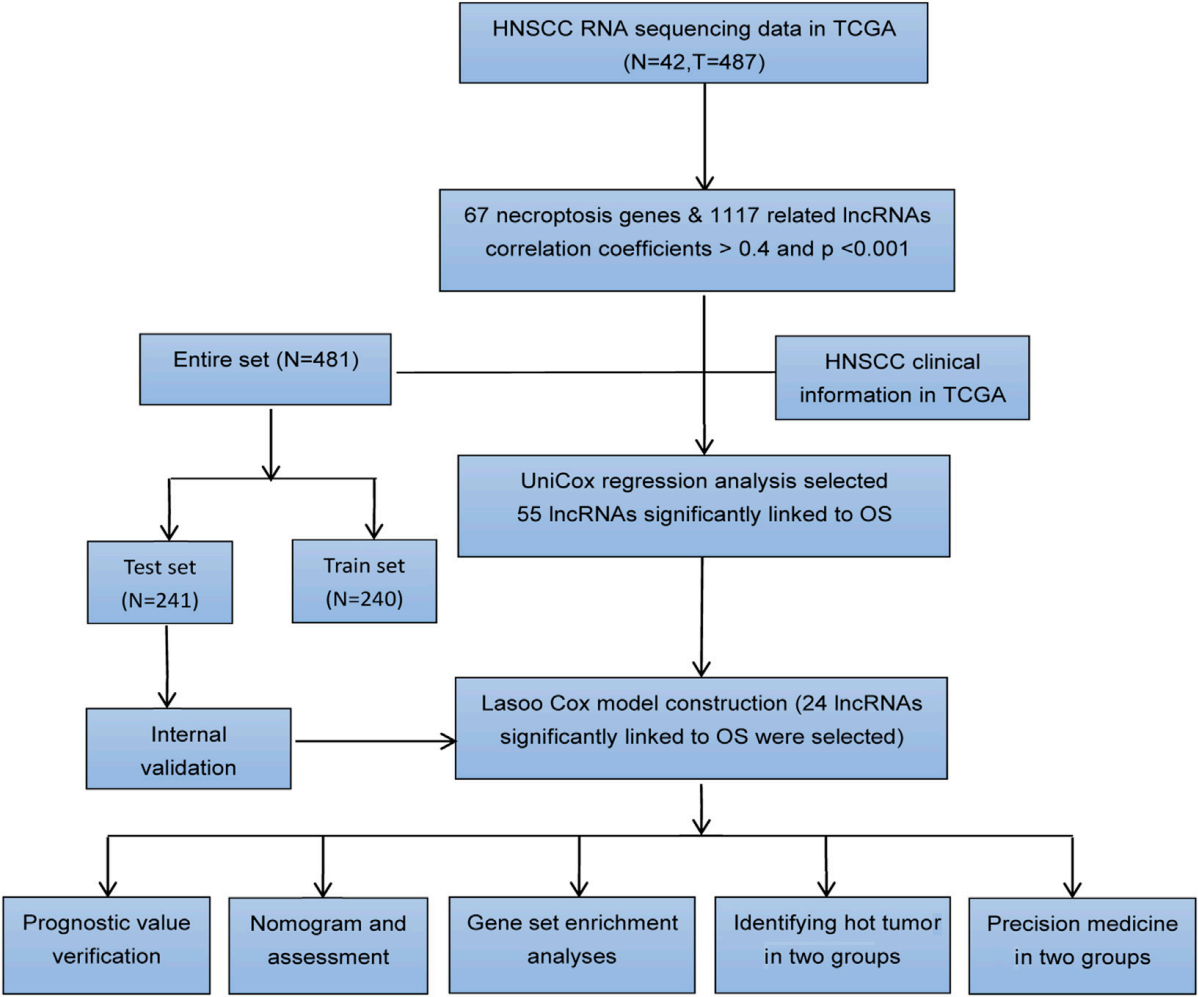
Workflow diagram of data analysis.

**FIGURE 2:**
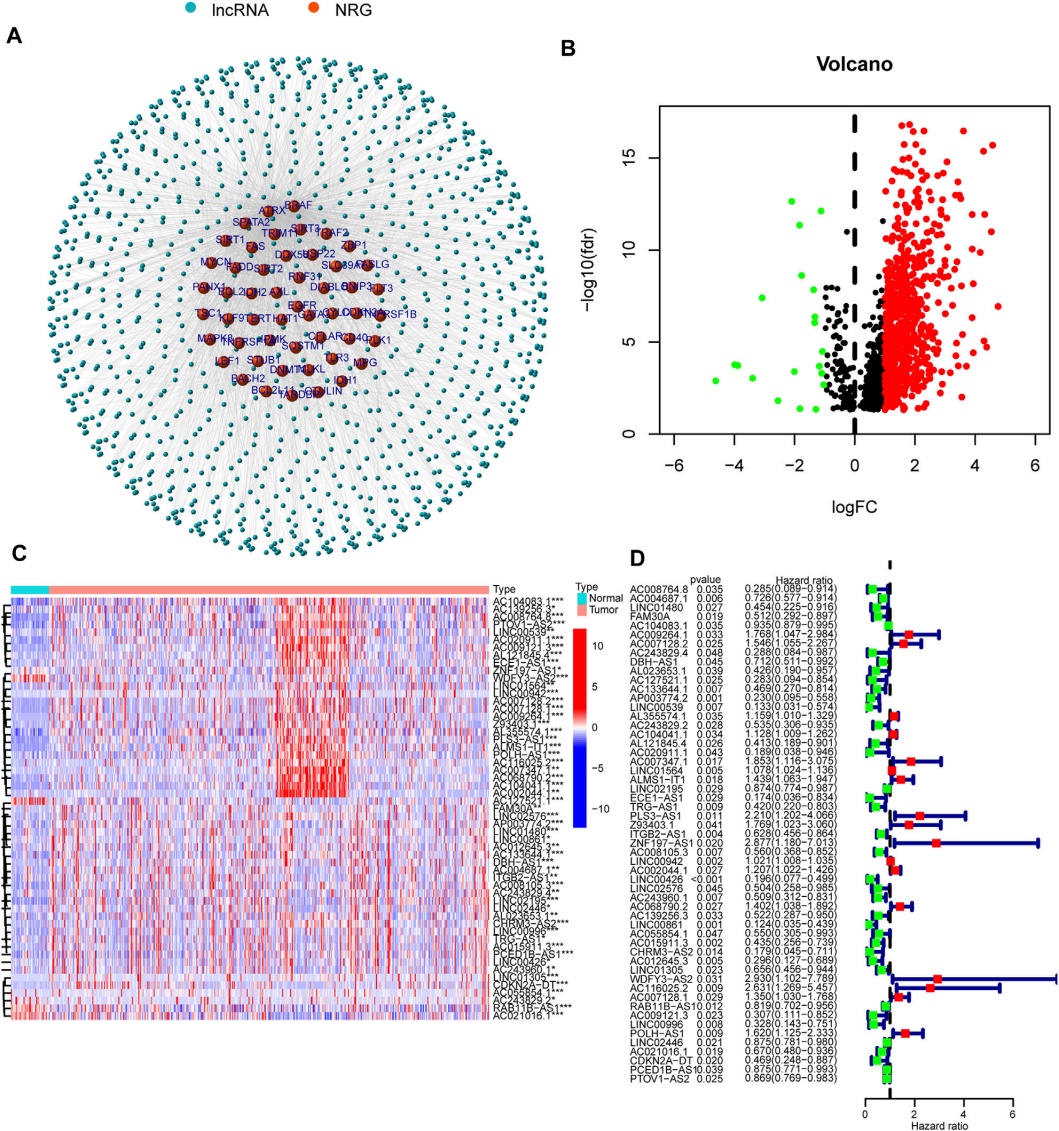
Necroptosis-related lncRNA prognostic signature identified in HNSCC. **(A)** Correlation network of necroptosis-related genes and necroptosis-related lncRNAs. **(B)** The volcano plot of 717 lncRNAs. Red dots represented upregulated lncRNAs and black dots represented down-regulated lncRNAs. **(C,D)** The expression and univariate Cox regression of 55 prognostic lncRNAs. *p < 0.05; **p < 0.01; ***p < 0.001.

Figure 1 shows the flow diagram depicting the present study. We compared the expression levels of 67 necroptosis-associated genes (Supplementary Table S1) between 42 healthy samples and 487 HNSCC cancer samples from the TCGA data and identified 14,086 lncRNAs. Among these lncRNAs, 1,117 lncRNAs met the criteria (Pearson correlation coefficients >0.4 and *p* values <0.001) (Figure 2A). We identified 717 differentially expressed necroptosis-related lncRNAs (|Log2FC| > 1 and *p* < 0.05) (Figure 2B); 697 were upregulated and 20 were downregulated. Univariate Cox regression showed 55 lncRNAs were significantly correlated with OS (*p* < 0.05 for all) (Figures 2C,D).

### Risk Model Construction and Verification

**FIGURE 3:**
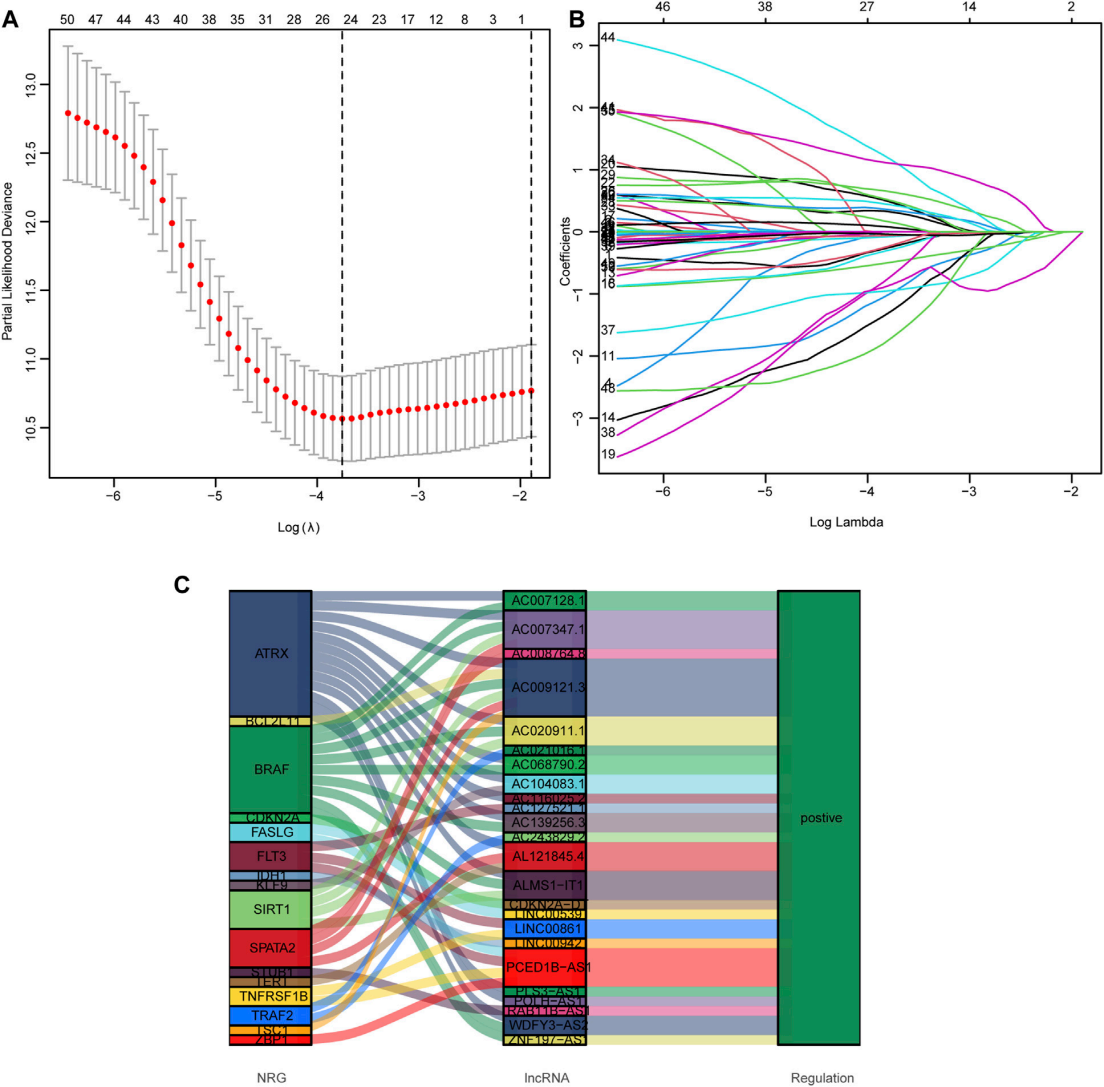
Construction of prognostic signature in HNSCC. **(A)** LASSO with 10-fold cross-validation. **(B)** Coefficient profile plots. **(C)** The Sankey diagram shows the connection degree between the 24 prognostic lncRNAs and the necroptosis-related genes.

To avoid overfitting and to quantify the impact of necroptosis-related lncRNAs on the prognosis of each HNSCC patient, we constructed a 24 lncRNAs prognostic signature by LASSO regression analysis (Figures 3A,B). All 24 lncRNAs positively regulated necroptosis genes in the Sankey diagram (Figure 3C).

We established the following formula to calculate the risk score of every HNSCC patient.

Risk score = AC008764.8×(-0.2393)+AC104083.1×(-0.0743)+AC127521.1×(-0.9739)+LINC00539×(-1.2625)+AC243829.2×(-0.4868)+AL121845.4×(-0.2809)+AC020911.1×(-0.6943)+AC007347.1×(0.4674)+ALMS1-IT1×(0.6387)+PLS3-AS1×(0.3155)+ZNF197-AS1×(0.4949)+LINC00942×(0.0017)+AC068790.2×(0.3482)+AC139256.3×(-0.9441)+LINC00861×(-0.8428)+WDFY3-AS2×(1.1448)+AC116025.2×(1.0996)+AC007128.1×(0.0713)+RAB11B-AS1×(-0.0588)+AC009121.3× (-1.6935)+POLH-AS1×(0.4292)+AC021016.1×(-0.0412)+CDKN2A-DT×(-0.2129)+PCED1B-AS1×(0.1493) (Meng et al., 2019).

**FIGURE 4:**
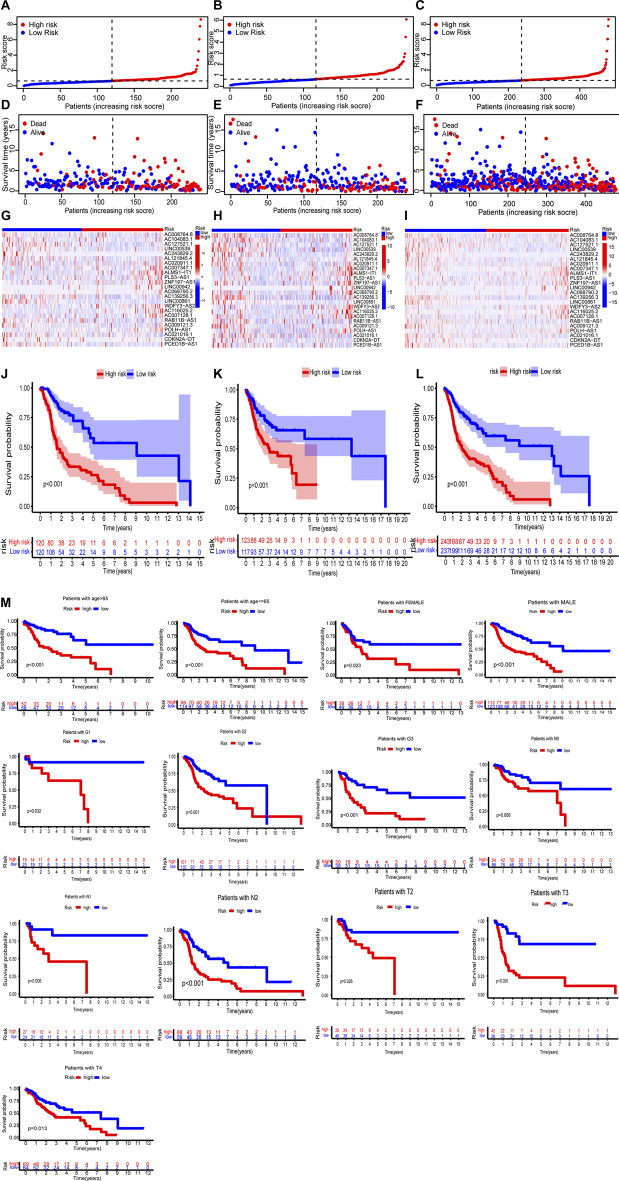
The prognosis analysis of the three sets (training set: A, D, G, J; test set: B, E, H, K; entire set: C, F, I, L). **(A–C)** Risk scores of the high-risk and low-risk groups in the respective three sets. **(D–F)** Comparison of survival between the two groups in the respective three sets. **(G–I)** The heat maps of 24 lncRNAs. **(J–L)** Kaplan–Meier survival curves of patients in the two groups in the respective three sets. **(M)** Kaplan–Meier survival curves of OS stratified by clinicopathologic parameters between the two groups.

In the training set, test set and entire set, the distribution of risk scores and survival times were compared between the high-risk group and the low-risk group (Figures 4A–C). More patients died in the high-risk group (Figures 4D–F). The heat maps of 24 lncRNAs are shown in Figures 4G–I. Survival curves show the high-risk groups of the three sets had poor prognoses (Figures 4J–L). Besides, some typical clinicopathologic parameters were identified to be prognostic factors (Figure 4M).

### Nomogram

**FIGURE 5:**
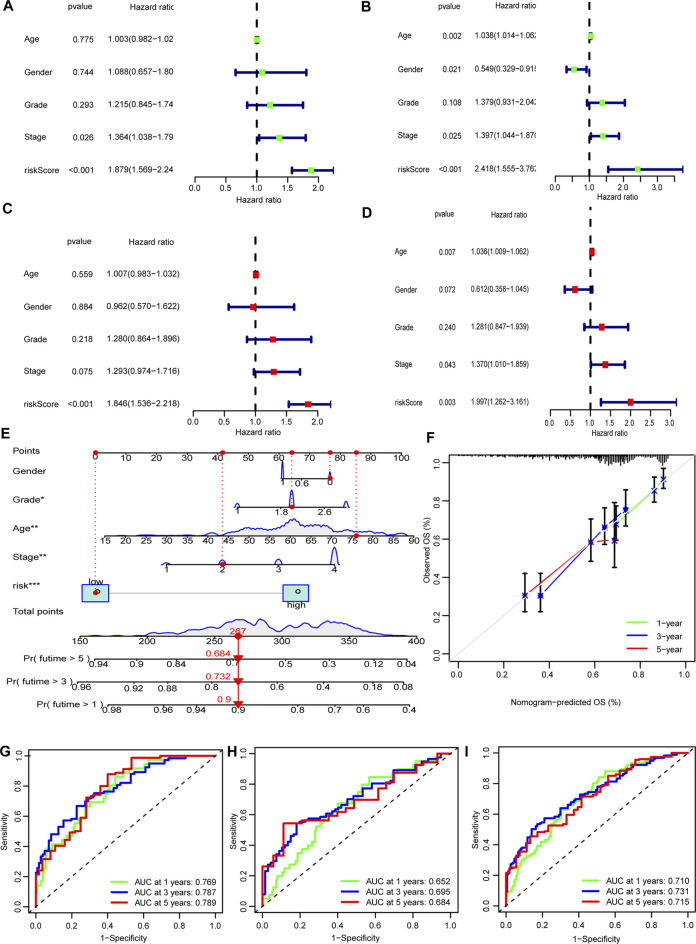
Nomogram of the model. **(A,B)** Tumor stage and risk score were risk factors in the training set. **(C,D)** Only risk score was risk factor in the test sets. **(E)** The nomogram that integrated the risk score, age, and tumor stage to predict OS. **(F)** The calibration curves for OS. **(G–I)** ROC curves of the three sets (training set: A, C, G; test set: B, D, H; entire set: I).

In both the training set and the test set, risk score, age and tumor stage were identified to be independent prognostic factors. The hazard ratios (HR) of these factors are shown in Figures 5A–D). A nomogram was established to predict the 1-, 3-, and 5-years OS (Figure 5E). The predicted survival showed close agreement with observed actual survival (Figure 5F).

### Assessment of the Risk Model

The ROC curves showed the sensitivity and specificity of the model were high (Figures 5G–I).

### GSEA

**FIGURE 6:**
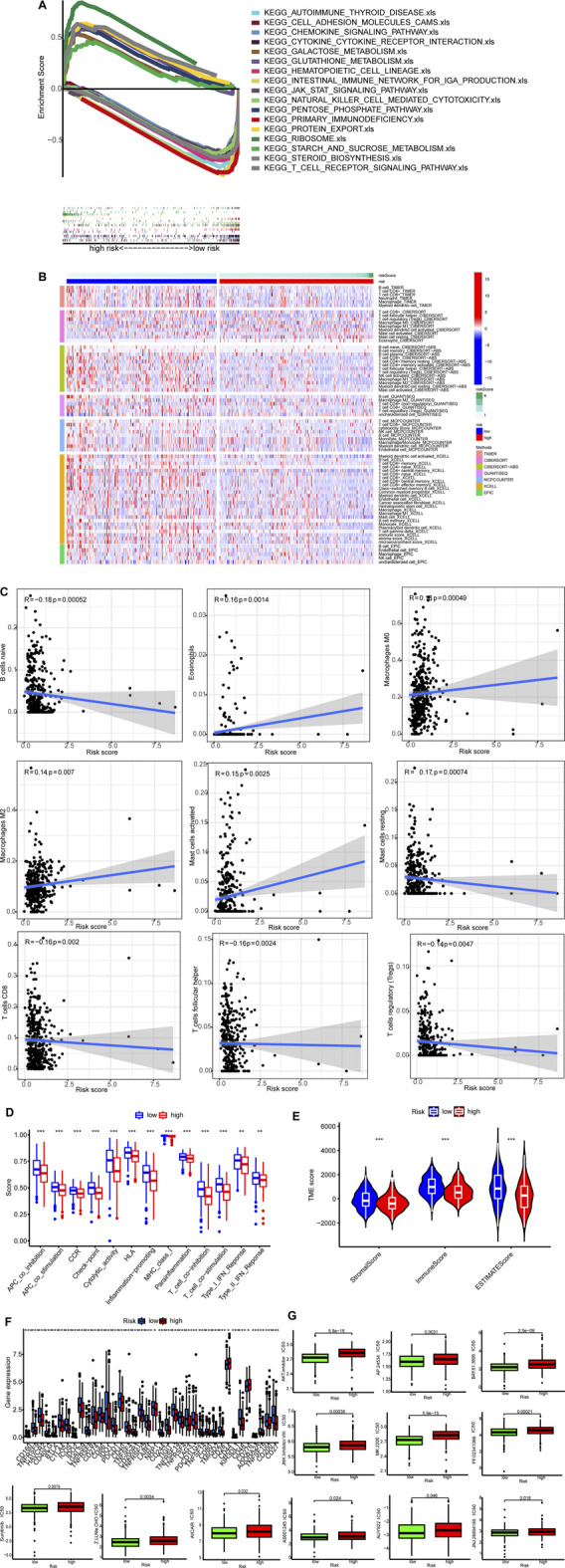
TME and immunotherapy. **(A)** Top 10 pathways identified by GSEA **(B)**The heat maps of immune cells. **(C)** Risk scores were corrected with immune cells. **(D)** ssGSEA scores of immune functions. **(E)** Comparison of immune-related scores between the two groups. **(F)** The expression of 34 checkpoints. **(G)**Twelve targeted and immunotherapeutic drugs with different IC50 between the low-risk group (green) and the high-risk group (red).

*GSEA* results showed that nine of top ten pathways were involved in carcinogenesis. For example, pentose and glucuronate interconversions, aldarate metabolism, and starch and sucrose metabolism were significantly enriched in the high-risk group. On the other hand, eight pathways enriched in the low-risk group were related to immunity (*p* < 0.05; FDR <0.25; |NES| > 1.9), such as T cell receptor signaling pathway and natural killer (NK) cell-mediated cytotoxicity (Figure 6A; Supplementary Table S2). Therefore, the low-risk group had a favorable TME. On the contrary, the high-risk group had an unfavorable TME.

### Cold and Hot Tumors

Single sample GSEA (ssGSEA) was performed to calculate numbers for different types of immune cells. Tumors of the low-risk group were infiltrated by more immune cells as exhibited in the heatmap (*p* < 0.05 for all) (Figure 6B; Supplementary Table S3). Correlations between risk scores and activities of immune cell types are shown in Figure 6C. All of the 13 immune-related pathways had higher activity in the low-risk group (Figure 6D).

ESTIMATE was used to generate immune scores and stromal scores. Figure 6E shows both immune scores and stromal scores (microenvironment) were higher in the low-risk group. Besides, the immune checkpoint expression was lower in the low-risk group (Figure 6F).

Finally, we found IC50 of the anti-tumor compounds, such as AKT inhibitors, JNK inhibitor and sunitinib, was usually lower in the low-risk group (Figure 6G).

## Discussion

The human genome produces a large amount of RNA transcripts that do not encode for proteins (Djebali et al., 2012). lncRNAs are among those transcripts. They are usually longer than 200 nucleotides and have many functions, including regulating cancer development (Huarte, 2015; Marchese et al., 2017; Mattick, 2018).

Necroptosis is mediated by RIP1 and RIP3 (Chan and Baehrecke, 2012; Pasparakis and Vandenabeele, 2015). RIP1 phosphorylates RIP3, which phosphorylates MLKL. In necrosomes phosphorylation of MLKL leads to MLKL oligomerization. Oligomerized MLKL causes cell death by breaking down cell membranes (Sun et al., 2012; Guicciardi et al., 2013). Although necroptosis may cause cancer cell death, cell death may inhibit immune response (Pasparakis and Vandenabeele, 2015; Wang et al., 2017). Necroptosis may elicit necrosis-associated inflammation. Inflammation could contribute to progression of cancer and promote resistance to anticancer treatments. In addition necroptosis may also fail to elicit strong immunogenic reactions.

Massively parallel RNA sequencing has identified large amounts of novel lncRNAs. However, functional annotation of lncRNAs is lagging behind. In the present study, we explored the prognostic values of necroptosis-related lncRNAs in HNSCC. We found that several necroptosis-related lncRNAs were closely related to HNSCC prognosis. More specifically, AC007347.1, ALMS1-IT1, PLS3-AS1, ZNF197-AS1, AC068790.2, WDFY3-AS2, AC116025.2, POLH-AS1, and PCED1B-AS1 were risk factors. On the other hand, AC008764.8, AC127521.1, LINC00539, AC243829.2, AL121845.4, AC020911.1, AC139256.3, LINC00861, AC009121.3 and CDKN2A-DT were protective factors for HNSCC patients.

Further analysis showed that AC007347.1, ALMS1-IT1, PLS3-AS1, ZNF197-AS1, AC068790.2, WDFY3-AS2, AC116025.2, POLH-AS1, and PCED1B-AS1 are positive regulators of BRAF, SIRT1, FLT3, FASLG, TRAF2, ATRX, TERT, SPATA2, and TNFRSF1B. BRAF is a proto-oncogene that encodes for the B-Raf protein, a kinase of the RAF protein family (Rebocho and Marais, 2013). The Ras/Raf/MAPK pathway regulates cell growth, differentiation, cell motility and apoptosis (Rebocho and Marais, 2013; Schettini et al., 2018). Abnormal activation of the pathway is responsible for many tumors (Bouchè et al., 2021).

SIRT1 is a member of the HDAC family. Aberrant SIRT1 expression has been found in many tumors (Bradbury et al., 2005; Hida et al., 2007; Stünkel et al., 2007; Chen et al., 2014). ATRX is a member of the SWI-SNF protein family (Stayton et al., 1994; Picketts et al., 1996; Argentaro et al., 2007). SWI-SNF proteins are involved in DNA recombination and repair (Picketts et al., 1996), which are crucial for both development and cancer (Watson et al., 2015). SPATA2 is a TNF receptor modulator. TNF-α pathway modulates immune responses (Swann et al., 2008). TNF-α and IL-1β induced SPATA2 expression in ovarian cancer cells and that increased SPATA2 expression was associated with poor prognosis of ovarian cancer patients (Wieser et al., 2019). Our study suggested SPATA2 expression is also associated with poor prognosis of HNSCC patients. ZBP1 is expressed in many tissues (Fu et al., 1999; Rothenburg et al., 2002) and is a interferon stimulated gene (Fu et al., 1999; Kuriakose and Kanneganti, 2018). ZBP1 expression in tumors is elevated. ZBP1 deletion blocks tumor necroptosis during tumor development and inhibits tumor metastasis (Baik et al., 2021). TNF-α is a pro-inflammatory cytokine mainly secreted by macrophages. There are two receptors for TNF-α, i.e., TNFRSF1A and TNFRSF1B. Although TNF can kill tumor cells, it also contribute to tumorigenesis (Aggarwal, 2003).

On the other hand, AC008764.8, AC127521.1, LINC00539, AC243829.2, AL121845.4, AC020911.1, AC139256.3, LINC00861, AC009121.3 and CDKN2A-DT were protective factors for HNSCC patients. Further analysis showed these lncRNAs were positive regulators of p16^INK4a^, SPATA2, FLT3, FASLG, TRAF2, ATRX, TERT, BRAF, SIRT1, TNFRSF1B, and BCL2L11. p16^INK4a^ is a tumor suppressor protein encoded by CDKN2A (Witcher and Emerson, 2009). p16^INK4a^ is a negative regulator of cell cycle (Serrano et al., 1993). CDKN2A also encodes for another tumor suppressor protein, which interacts with p53 (Pomerantz et al., 1998). Inactivation of p16^INK4a^ has been observed in various cancers *via* various mechanisms (Zhao et al., 2016). FLT3 is a receptor tyrosine kinase that is expressed in hematopoietic cells. Activation of FLT3 leads to autophosphorylation and mediates proliferation and differentiation of hematopoietic progenitor cells. However its role in tumorogenesis has not been reported. FASLG is a tumor suppressor and a member of the tumor necrosis factor superfamily (Magerus et al., 2021). FASLG/FAS signaling could induce apoptosis in various cancers (Liu et al., 2009; Kadam and Abhang, 2016; Magerus et al., 2021). TRAFs are intracellular adaptor signaling molecules of immune cells (Rothe et al., 1995; Ye et al., 2002; Park, 2018). TRAF2 promotes p53-dependent apoptosis by activating the JNK signaling cascade in cancer cells (Tsuchida et al., 2020). BCL2L11 is a member of BCL-2 family and regulates function of mitochondria (Concannon et al., 2010; Kilbride et al., 2010). BCL2L11 deletion/downregulation is found in many neoplasms and contribute to acquired drug resistance (Zhang et al., 2016).

By our model, we found pathways such as TNF, RAF and BCL-2 and FASLG/FAS are closely related to HNSCC. Although the protective lncRNAs are positive regulators of several tumor suppressors, they are also associated with several oncogenes. We propose that the prognostic value of a specific lncRNA is determined by the net effect of its multiple target genes.

Tumors have been described as “hot” or “cold” according to infiltration degree by T cells rushing to fight the cancerous cells. Hot tumors typically respond well to immunotherapy treatment using checkpoint inhibitors. Checkpoint inhibitors block signalling through checkpoint receptors to prevent the loss of T cell response to tumors. In contrast, nonimmunogenic “cold” tumors have not yet been infiltrated with T cells. The lack of T cells makes it difficult to provoke an immune response with immunotherapy drugs. IN addition, the microenvironment surrounding cold tumors contains myeloid-derived suppressor cells and T regulatory cells, which are known to dampen the immune response. In our model the patients in the high-risk group were more likely to have cold tumors, which may partially explain why the patients in the high-risk group had poor prognosis.

There were some limitations of our model. As a retrospective study, inherent biases might affect the model. We had performed internal validation by the test set, but we did not perform external validation.

In conclusion, we established a novel necroptosis-associated lncRNA signature for the prognosis of HNSCC. The established signatures suggest that lncRNAs might be associated with responses to targeted therapy and immunotherapy of HNSCC. The potential of this signature in predicting patient survival and treatment responses need to be validated in future tests.

## Data Availability

The original contributions presented in the study are included in the article/Supplementary Material, further inquiries can be directed to the corresponding authors.
