# Supplementary material for: A Novel Necroptosis-Associated IncRNAs Signature for Prognosis of Head and Neck Squamous Cell Carcinoma
Source: Front Genet. 2022 Jun 8;13:907392. doi: 10.3389/fgene.2022.907392 (PMC9213787; doi:10.3389/fgene.2022.907392)
Supplement: Supplementary file 2 [file Table2.DOCX]

Tables S3. GSEA report of pathways significantly enriched in high and low risk groups.

| **NAME** | **SIZE** | **ES** | **NES** | **NOM p-val** | **FDR q-val** |
| --- | --- | --- | --- | --- | --- |
| KEGG PENTOSE AND GLUCURONATE INTERCONVERSIONS | 28 | 0.62816423 | 1.8056114 | 0.00996016 | 0.28722543 |
| KEGG PENTOSE PHOSPHATE PATHWAY | 27 | 0.60186315 | 1.7688421 | 0.014830508 | 0.18853387 |
| KEGG GALACTOSE METABOLISM | 26 | 0.5342091 | 1.6485666 | 0.018518519 | 0.23302686 |
| KEGG GLUTATHIONE METABOLISM | 49 | 0.58871603 | 1.815458 | 0.018789144 | 0.53731227 |
| KEGG PROTEIN EXPORT | 24 | 0.63367254 | 1.7269344 | 0.028513238 | 0.19854599 |
| KEGG STARCH AND SUCROSE METABOLISM | 52 | 0.45926198 | 1.5995461 | 0.02952756 | 0.23640972 |
| KEGG RIBOSOME | 88 | 0.83999985 | 1.7919409 | 0.030674847 | 0.21366197 |
| KEGG STEROID BIOSYNTHESIS | 17 | 0.6318807 | 1.603542 | 0.049309663 | 0.2606866 |

| **NAME** | **SIZE** | **ES** | **NES** | **NOM p-val** | **FDR q-val** |
| --- | --- | --- | --- | --- | --- |
| KEGG T CELL RECEPTOR SIGNALING PATHWAY | 108 | -0.6720114 | -2.3173528 | 0 | 7.01E-04 |
| KEGG JAK STAT SIGNALING PATHWAY | 155 | -0.6154994 | -2.2994895 | 0 | 7.71E-04 |
| KEGG INTESTINAL IMMUNE NETWORK FOR IGA PRODUCTION | 46 | -0.85123485 | -2.2861788 | 0 | 0.001028944 |
| KEGG NATURAL KILLER CELL MEDIATED CYTOTOXICITY | 132 | -0.634536 | -2.277166 | 0.004040404 | 9.82E-04 |
| KEGG FC EPSILON RI SIGNALING PATHWAY | 79 | -0.62056917 | -2.2748775 | 0 | 7.86E-04 |
| KEGG CYTOKINE CYTOKINE RECEPTOR INTERACTION | 264 | -0.6371375 | -2.2409608 | 0.002096436 | 9.71E-04 |
| KEGG AUTOIMMUNE THYROID DISEASE | 50 | -0.77297205 | -2.1890602 | 0.002070393 | 0.002394127 |
| KEGG CHEMOKINE SIGNALING PATHWAY | 188 | -0.62336206 | -2.188913 | 0.001926782 | 0.002094861 |
| KEGG PRIMARY IMMUNODEFICIENCY | 35 | -0.83170265 | -2.1663942 | 0 | 0.002823275 |
| KEGG HEMATOPOIETIC CELL LINEAGE | 85 | -0.7040948 | -2.1531942 | 0.002020202 | 0.002695606 |
| KEGG CELL ADHESION MOLECULES CAMS | 131 | -0.66867113 | -2.1441715 | 0.001988072 | 0.002815196 |
| KEGG ASTHMA | 28 | -0.87921053 | -2.1397479 | 0 | 0.002580596 |
