# Supplementary material for: A Novel Necroptosis-Associated IncRNAs Signature for Prognosis of Head and Neck Squamous Cell Carcinoma
Source: Front Genet. 2022 Jun 8;13:907392. doi: 10.3389/fgene.2022.907392 (PMC9213787; doi:10.3389/fgene.2022.907392)
Supplement: Supplementary file 3 [file Table3.DOCX]

Tables S4. The immune cells in risk groups .

| **immune** | **pvalue** |
| --- | --- |
| B cell_TIMER | 5.94E-08 |
| T cell CD4+_TIMER | 8.00E-06 |
| T cell CD8+_TIMER | 0.000523212 |
| Neutrophil_TIMER | 2.34E-06 |
| Macrophage_TIMER | 0.007701002 |
| Myeloid dendritic cell_TIMER | 7.05E-05 |
| T cell CD8+_CIBERSORT | 2.28E-06 |
| T cell follicular helper_CIBERSORT | 3.23E-05 |
| T cell regulatory (Tregs)_CIBERSORT | 0.010613996 |
| Macrophage M0_CIBERSORT | 0.008977995 |
| Macrophage M1_CIBERSORT | 8.86E-05 |
| Myeloid dendritic cell activated_CIBERSORT | 0.002468769 |
| Mast cell activated_CIBERSORT | 0.006335697 |
| Mast cell resting_CIBERSORT | 1.83E-05 |
| Eosinophil_CIBERSORT | 2.91E-05 |
| B cell naive_CIBERSORT-ABS | 0.006400192 |
| B cell memory_CIBERSORT-ABS | 0.028983732 |
| B cell plasma_CIBERSORT-ABS | 0.011729733 |
| T cell CD8+_CIBERSORT-ABS | 4.55E-13 |
| T cell CD4+ memory resting_CIBERSORT-ABS | 0.00992463 |
| T cell CD4+ memory activated_CIBERSORT-ABS | 0.010780682 |
| T cell follicular helper_CIBERSORT-ABS | 1.17E-15 |
| T cell regulatory (Tregs)_CIBERSORT-ABS | 2.60E-06 |
| NK cell activated_CIBERSORT-ABS | 1.14E-05 |
| Macrophage M1_CIBERSORT-ABS | 8.28E-11 |
| Macrophage M2_CIBERSORT-ABS | 9.45E-07 |
| Myeloid dendritic cell resting_CIBERSORT-ABS | 0.002064455 |
| Mast cell activated_CIBERSORT-ABS | 0.000712772 |
| B cell_QUANTISEQ | 1.04E-08 |
| Macrophage M2_QUANTISEQ | 7.61E-05 |
| T cell CD4+ (non-regulatory)_QUANTISEQ | 0.001786614 |
| T cell CD8+_QUANTISEQ | 1.23E-06 |
| T cell regulatory (Tregs)_QUANTISEQ | 2.23E-07 |
| uncharacterized cell_QUANTISEQ | 0.002040978 |
| T cell_MCPCOUNTER | 1.41E-06 |
| T cell CD8+_MCPCOUNTER | 3.87E-08 |
| cytotoxicity score_MCPCOUNTER | 0.000325279 |
| NK cell_MCPCOUNTER | 4.22E-08 |
| B cell_MCPCOUNTER | 2.94E-09 |
| Monocyte_MCPCOUNTER | 0.007595204 |
| Macrophage/Monocyte_MCPCOUNTER | 0.007595204 |
| Myeloid dendritic cell_MCPCOUNTER | 1.55E-10 |
| Endothelial cell_MCPCOUNTER | 0.025450127 |
| Myeloid dendritic cell activated_XCELL | 1.14E-05 |
| B cell_XCELL | 1.79E-07 |
| T cell CD4+ memory_XCELL | 0.033540764 |
| T cell CD4+ naive_XCELL | 3.44E-08 |
| T cell CD4+ central memory_XCELL | 0.000324478 |
| T cell CD8+ naive_XCELL | 0.009900993 |
| T cell CD8+_XCELL | 5.43E-10 |
| T cell CD8+ central memory_XCELL | 1.19E-07 |
| T cell CD8+ effector memory_XCELL | 3.49E-07 |
| Class-switched memory B cell_XCELL | 8.62E-09 |
| Common myeloid progenitor_XCELL | 0.046065601 |
| Myeloid dendritic cell_XCELL | 0.000565052 |
| Endothelial cell_XCELL | 0.006606251 |
| Cancer associated fibroblast_XCELL | 0.000724794 |
| Hematopoietic stem cell_XCELL | 0.000241313 |
| Macrophage_XCELL | 0.027595588 |
| Macrophage M1_XCELL | 0.013760662 |
| Mast cell_XCELL | 0.000290886 |
| B cell memory_XCELL | 0.000510208 |
| Monocyte_XCELL | 0.032732858 |
| Plasmacytoid dendritic cell_XCELL | 4.73E-06 |
| T cell gamma delta_XCELL | 0.017099723 |
| immune score_XCELL | 1.75E-09 |
| stroma score_XCELL | 0.001003462 |
| microenvironment score_XCELL | 2.44E-10 |
| B cell_EPIC | 2.91E-05 |
| Endothelial cell_EPIC | 0.001625246 |
| Macrophage_EPIC | 0.000759305 |
| NK cell_EPIC | 0.007565485 |
| uncharacterized cell_EPIC | 0.000133429 |
